# Mendelian randomization analysis of the association between human blood cell traits and uterine polyps

**DOI:** 10.1038/s41598-021-84851-0

**Published:** 2021-03-04

**Authors:** Shuliu Sun, Yan Liu, Lanlan Li, Minjie Jiao, Yufen Jiang, Beilei Li, Wenrong Gao, Xiaojuan Li

**Affiliations:** Department of Obstetrics and Gynecology, Northwest Women’s and Children’s Hospital, Xi’an, 710061 Shaanxi China

**Keywords:** Biomarkers, Medical research, Signs and symptoms

## Abstract

Human blood cells (HBCs) play essential roles in multiple biological processes but their roles in development of uterine polyps are unknown. Here we implemented a Mendelian randomization (MR) analysis to investigate the effects of 36 HBC traits on endometrial polyps (EPs) and cervical polyps (CPs). The random-effect inverse-variance weighted method was adopted as standard MR analysis and three additional MR methods (MR-Egger, weighted median, and MR-PRESSO) were used for sensitivity analyses. Genetic instruments of HBC traits was extracted from a large genome-wide association study of 173,480 individuals, while data for EPs and CPs were obtained from the UK Biobank. All samples were Europeans. Using genetic variants as instrumental variables, our study found that both eosinophil count (OR 0.85, 95% CI 0.79–0.93, P = 1.06 × 10^−4^) and eosinophil percentage of white cells (OR 0.84, 95% CI 0.77–0.91, P = 2.43 × 10^−5^) were associated with decreased risk of EPs. The results were robust in sensitivity analyses and no evidences of horizontal pleiotropy were observed. While we found no significant associations between HBC traits and CPs. Our findings suggested eosinophils might play important roles in the pathogenesis of EPs. Besides, out study provided novel insight into detecting uterine polyps biomarkers using genetic epidemiology approaches.

## Introduction

Polyps are frequently observed pathological growths in the uterus that occur in women during of both reproductive and postmenopausal age^1^. These structures are categorized based on their size, number, location, and presence/absence of a stalk. Endometrial polyps (EPs) are the most commonly diagnosed type of uterine polyps, with an estimated prevalence ranging from 7.8 to 50%, while cervical polyps (CPs) are the second most common (with an estimated prevalence of 2–5%)^2–4^. The polyps are usually asymptomatic but may cause a number of problems, such as abnormal uterine bleeding, subfertility, and risk of malignancy^5–9^. A number of etiological theories have suggested an association between the pathogenesis of EPs and CPs and various factors, including estrogen overstimulation, chronic inflammation, and genetic predisposition^5,10,11^. However, early biomarkers for informing diagnosis and identifying pathological mechanisms are still lacking.

Human blood cells (HBCs) play essential roles in oxygen transport, hemostasis, osmotic regulation, and clearance of necrotic tissue and toxins, and are involved in multiple inflammatory and immune responses of the human body^12–16^. Changes in HBC traits may indicate disturbances in physiological processes and are associated with a series of pathological structural abnormalities, such as cellular degeneration, tissue proliferation, and even tumor formation^17,18^. In fact, chronic inflammation is a known etiological factor for both EPs and CPs, and histopathological examinations have shown that polyps are consistently accompanied by inflammatory infiltration^1,5^. This indicates that HBCs might participate in important biological processes during the development of uterine polyps. However, the associations between HBC traits and uterine polyps have not been investigated.

Mendelian randomization (MR) is a novel study design that uses genetic variants as instrumental variables (IVs) to investigate the relationship between risk factors and clinical outcomes of interest^19^. The fundamental principle utilized in the MR design is that if genetic variants could predict a certain proportion of variance for a modifiable exposure, then they should be also causally associated with an exposure-related disease risk. MR presents a number of advantages over traditional observational studies, including the ability to prevent confounding, reverse causation, and various biases that are common in observational epidemiological studies^20^. Recently, the explosion in publicly available summary statistics of genome-wide association studies (GWASs) has provided extensive resources for the application of MR^21^. Here, by extracting summary data from a GWAS of HBC traits and uterine polyps, our study aims to provide an unbiased investigation of the effects of HBC traits on EPs and CPs.

## Results

### Strength of IVs

**Figure 1:**
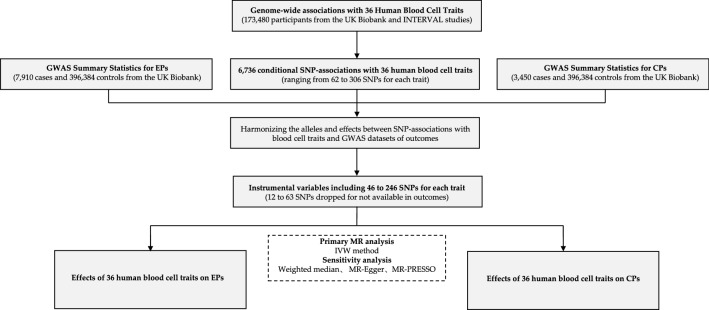
Schematic representation of the study design.

After harmonizing the alleles and effects between single nucleotide polymorphisms (SNP) associations with blood cell traits and GWAS datasets of outcomes, we obtained 46–246 genome-wide SNPs for the 36 HBC traits (Fig. 1; Supplementary Tables 1, 2). On average, the SNPs explained 10.8% (in the range of 2.8–28.3%) of the variance in their corresponding HBC traits. The median F statistic, another parameter for measuring the strength of IVs, was 114.2 (in the range of 75.9–281.9), meaning that all IVs were strong (the recommended F statistic is > 10) for the MR analyses.

### Effects of HBC traits on EPs and CPs

**Figure 2:**
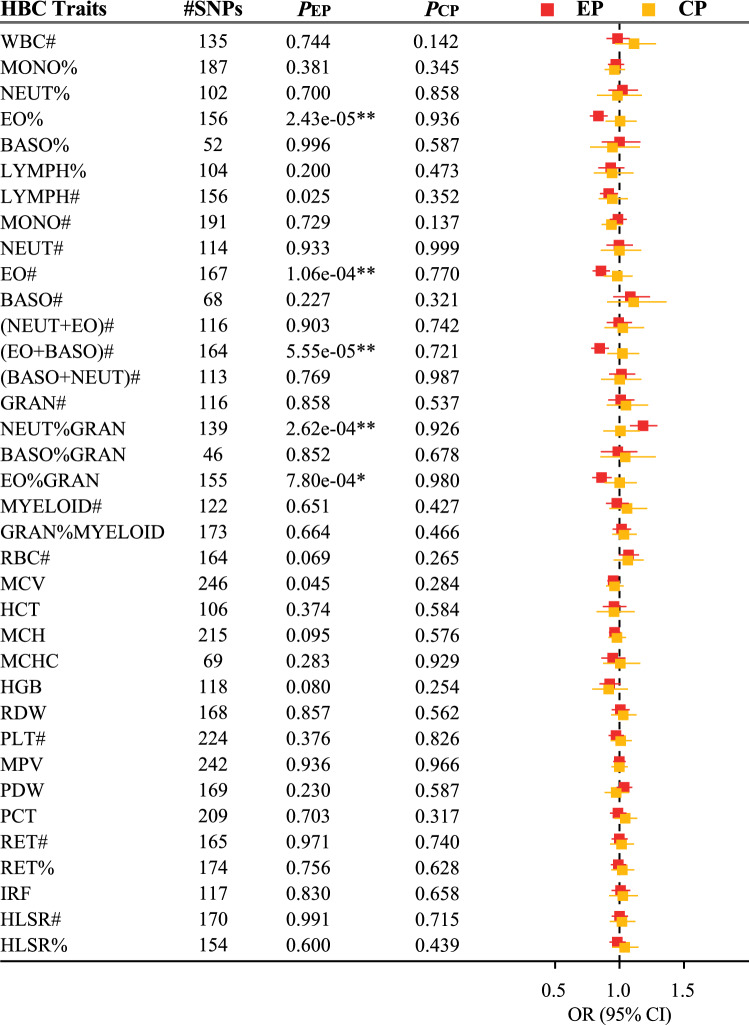
Mendelian randomization estimates of HBC traits on EPs and CPs. Associations were assessed using the random-effect IVW method. Results are expressed as ORs and 95% CIs per 1 SD of each HBC trait. *HBC* human blood cell, *EPs* endometrial polyps, *CPs* cervical polyps, *IVW* inverse-variance weighted. **Multiple-testing-adjusted threshold: *P* < 6.94 × 10^–4^. *Suggestive for association: *P* < 0.01.

Primary results of MR estimates are presented in Fig. 2. Following the Bonferroni-corrected significance threshold (P < 6.94 × 10^–4^), the random-effect inverse-variance weighted (IVW) method identified four HBC traits that showed significant associations with EPs, including the eosinophil percentage of white cells (EO%; odds ratio [OR] 0.84; 95% Confidence interval [CI] 0.77–0.91; *P*_IVW_ = 2.43 × 10^−5^), eosinophil count (EO#; OR 0.85; 95% CI 0.79–0.93; *P*_IVW_ = 1.06 × 10^−4^), sum eosinophil basophil counts ([EO + BASO]#; OR 0.84; 95% CI 0.78–0.92; *P*_IVW_ = 5.55 × 10^−5^), and neutrophil percentage of granulocytes (NEUT%GRAN; OR 1.18; 95% CI 1.08–1.30; *P*_IVW_ = 2.62 × 10^−4^). All four traits were granulocyte-related, and three were related to eosinophils in particular. Notably, the three eosinophil-related traits were associated with a decreased risk of EPs (the ORs range from 0.84 to 0.85), while the NEUT%GRAN was associated with an increased risk of EPs. However, we found no evidence for associations between blood cell traits and CPs.

### Sensitivity analyses

Sensitivity analyses showed consistent results with the primary random-effect IVW estimates for all four HBC traits (Table 1), and no strong evidences of horizontal pleiotropy were observed (*P*_intercept_ = 0.787 for EO%, *P*_intercept_ = 0.774 for EO#, *P*_intercept_ = 0.513 for [EO + BASO]#, and *P*_intercept_ = 0.433 for NEUT%GRAN), indicating robust relationships between the four granulocyte-related traits and EPs (Table 1). We further investigated heterogeneity between genetic instruments used which could also indicate pleiotropic effects. However, all four MR associations between HBC traits and EPs presented some evidences of heterogeneity (I^2^ > 25% or Cochran Q-derived P < 0.1). To control for heterogeneity in these MR estimates, we further performed outliers-corrected MR analyses by removing weak or pleiotropic instruments detected by Cochran’s Q tests. After removing the identified outliers, the effects of the four HBC traits on EPs were still robust (OR [95% CI] 0.81[0.75,0.88] and *P*_IVW_ = 4.89 × 10^−7^ for EO%, OR [95% CI] 0.84[0.77,0.90] and *P*_IVW_ = 3.26 × 10^−6^ for EO#, OR [95% CI] 0.83[0.77,0.89] and *P*_IVW_ = 1.64 × 10^−6^ for [EO + BASO]#, OR [95% CI] 1.22[1.12,1.32] and *P*_IVW_ = 5.98 × 10^−6^ for NEUT%GRAN).

**Table 1:** MR estimates of associations between human blood cell (HBC) traits and endometrial polyps (Eps).

HBC trait	Methods	First-stage MR analyses				Outliers corrected MR analyses^b^			
		#SNPs	OR (95% CI)	P value	Heterogeneity test^a^	#SNPs	OR (95% CI)	P value	Heterogeneity test
EO%	Random-effect IVW	156	0.84 (0.77, 0.91)	2.43e−05	Q = 184.6 I^2^ = 16.0% P = 0.053	142	0.81 (0.75, 0.88)	4.89e−07	Q = 95.6 I^2^ = 0% P = 0.999
	Weighted median		0.81 (0.72, 0.92)	9.91e−04			0.81 (0.71, 0.91)	6.42e−04	
	MR-PRESSO		0.84 (0.77, 0.91)	4.13e−05			0.81 (0.76, 0.87)	9.29e−09	
	MR-Egger		0.86 (0.71, 1.03)	0.105			0.77 (0.65, 0.92)	0.004	
	MR-Egger (intercept)		–	0.787			–	0.527	
EO#	Random-effect IVW	167	0.85 (0.79, 0.93)	1.06e−04	Q = 197.2 I^2^ = 15.8% P = 0.049	154	0.84 (0.77, 0.90)	3.26e−06	Q = 123.9 I^2^ = 0% P = 0.959
	Weighted median		0.83 (0.70, 0.99)	0.049			0.80 (0.71, 0.90)	2.06e−04	
	MR-PRESSO		0.85 (0.79, 0.93)	1.52e−04			0.84 (0.78, 0.89)	7.79e−07	
	MR-Egger		0.81 (0.72, 0.92)	6.42e−04			0.76 (0.64, 0.90)	0.001	
	MR-Egger (intercept)		–	0.774			–	0.212	
(EO + BASO)#	Random-effect IVW	164	0.84 (0.78, 0.92)	5.55e−05	Q = 194.3 I^2^ = 16.1% P = 0.047	152	0.83 (0.77, 0.89)	1.64e−06	Q = 125.7 I^2^ = 0% P = 0.934
	Weighted median		0.80 (0.71, 0.91)	3.76e−04			0.80 (0.71, 0.91)	4.67e−04	
	MR-PRESSO		0.84 (0.78, 0.92)	8.50e−05			0.83 (0.77, 0.89)	4.98e−07	
	MR-Egger		0.80 (0.66, 0.96)	0.018			0.75 (0.63, 0.89)	9.04e−04	
	MR-Egger (intercept)		–	0.513			–	0.191	
NEUT%GRAN	Random-effect IVW	139	1.18 (1.08, 1.30)	2.62e−04	Q = 167.0 I^2^ = 17.3% P = 0.047	128	1.22 (1.12, 1.32)	5.98e−06	Q = 96.8 I^2^ = 0% P = 0.978
	Weighted median		1.23 (1.07, 1.41)	0.004			1.23 (1.08, 1.41)	0.002	
	MR-PRESSO		1.18 (1.08, 1.30)	3.71 e−04			1.22 (1.13, 1.31)	8.37e−07	
	MR-Egger		1.27 (1.04, 1.57)	0.021			1.35 (1.11, 1.63)	0.002	
	MR-Egger (intercept)		–	0.433			–	0.243	

*HBC* human blood cell, *MR* Mendelian randomization, *EO*# eosinophil count, *EO%* eosinophil percentage of white cells, *(EO + BASO)#* sum eosinophil basophil count, *NEUT%GRAN* neutrophil percentage of granulocytes, *#SNPs* number of single nucleotide polymorphisms, *IVW* inverse-variance weighted, *MR-PRESSO* MR-pleiotropy residual sum and outlier.

^a^Heterogeneity was assessed based on the Cochran’s Q statistic, quantified *I*^2^ index and Cochran’s Q-derived *P* value according to the IVW model.

^b^Outliers were detected using individual components of Cochran’s Q according to the IVW model. Secondary MR analyses were performed after removing the detected outliers.

### Multivariable MR analysis

**Figure 3:**
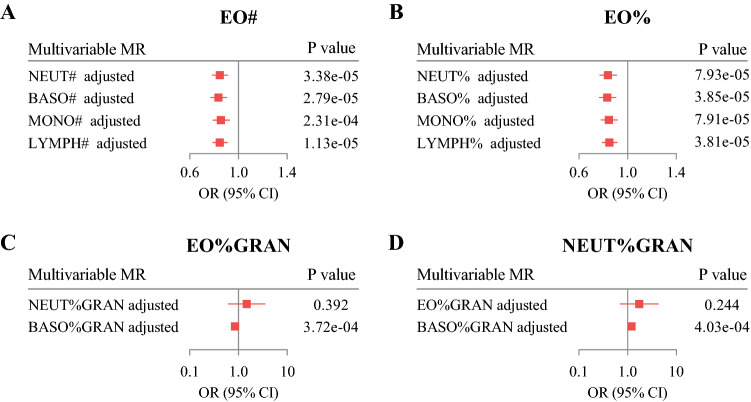
Multivariable MR analysis for four HBC traits adjusting for variables within the same category. (**A**) Effect of EO# on EPs, adjusting for NEUT#, BASO#, MONO#, and LYMPH#; (**B**) effect of EO% on EPs, adjusting for NEUT%, BASO%, MONO%, and LYMPH%; (**C**) effect of EO%GRAN on EPs, adjusting for NEUT%GRAN and BASO%GRAN; (**D**) effect of NEUT%GRAN on EPs, adjusting for EO%GRAN and BASO%GRAN. Results are expressed as ORs and 95% CIs per 1 SD of each HBC trait. *HBC* human blood cell, *EPs* endometrial polyps.

To control for bias introduced by genetic instrument overlaps among different blood cell traits, we performed multivariable MR analysis adjusting for variables within the same category. The effect of EO# on EP was robust adjusting for NEUT# (OR 0.85; 95% CI 0.78–0.91, P_IVW_ = 3.38 × 10^−5^), BASO# (OR 0.84; 95% CI 0.77–0.91, P_IVW_ = 2.79 × 10^−5^), MONO# (OR 0.86; 95% CI 0.79–0.93, P_IVW_ = 2.31 × 10^−4^), and LYMPH# (OR 0.85; 95% CI 0.79–0.91, P_IVW_ = 1.13 × 10^−5^) and the effect estimates were consistent with initial MR analysis (Fig. 3A). Similar results were also seen in effects of EO% on EPs (Fig. 3B). Notably, we found that there was interaction between NEUT%GRAN and EO%GRAN when performing multivariable MR on EPs (Fig. 3C,D). More than half of SNPs were overlapped between IVs for NEUT%GRAN (99 out of 139) and EO%GRAN (99 out of 155). Thus, the effect of NEUT%GRAN on EPs might be false positive and the real effect was caused by EO%GRAN changes, considering the fact that there was a shift in the relationship between EO%GRAN and NEUT%GRAN.

### Sub-study MR analysis

We further performed sub-study MR analysis by utilizing samples separately from the UK Biobank, UK BiLEVE and INTERVAL. The effect estimates of EO# on EPs were consistent (the ORs range from 0.85 to 0.86) across the three studies (Table 2). Similar results were also seen for EO% (the ORs range from 0.83 to 0.84) and (EO + BASO)# (the ORs range from 0.84 to 0.86), suggesting that the effects of EO#, EO% and were (EO + BASO)# robust and potential bias from sample overlaps could be ignored.

**Table 2:** Sub-study MR analysis of eosinophil properties on EPs.

Exposure	Study	Sample size	OR (95% CI)	P value
EO#	UK Biobank	87,265	0.86 (0.79, 0.93)	1.41e−04
	UK BiLEVE	45,694	0.86 (0.79, 0.93)	1.59e−04
	INTERVAL	40,521	0.86 (0.79, 0.93)	9.58e−05
	Combined	173,480	0.85 (0.79, 0.93)	1.06e−04
EO%	UK Biobank	87,265	0.84 (0.77, 0.91)	2.33e−05
	UK BiLEVE	45,694	0.84 (0.78, 0.92)	7.02e−05
	INTERVAL	40,521	0.84 (0.77, 0.91)	2.77e−05
	Combined	173,480	0.84 (0.77, 0.91)	2.55e−05
(EO + BASO)#	UK Biobank	87,265	0.85 (0.79, 0.93)	1.06e−04
	UK BiLEVE	45,694	0.85 (0.78, 0.92)	7.00e−05
	INTERVAL	40,521	0.85 (0.79, 0.92)	3.88e−05
	Combined	173,480	0.84 (0.78, 0.92)	5.55e−05

MR estimates were performed using samples separately from UK Biobank, UK BiLEVE and INTERVAL. Associations were assessed using the random-effect IVW method. Results are expressed as ORs and 95% CIs per 1 SD of each HBC trait.

*EPs* endometrial polyps, *IVW* inverse-variance weighted.

## Discussion

Our study provided valuable information for screening novel biomarkers and understanding the pathophysiological mechanisms of EPs and CPs. We identified three eosinophil-related properties that were robustly associated with EPs, suggesting that eosinophils might play important roles in the pathogenesis of EPs. While we found no significant associations between HBC traits and CPs.

The associations between eosinophil properties and EPs had not yet been reported prior to this study. Eosinophils are multifunctional granulocytes involved in the pathogenesis of diverse inflammatory processes, including parasitic infections and allergic reactions^22^. Activated eosinophils release a series of proteins, cytokines, chemokines, and lipid mediators that participate in multiple biological processes such as endothelial proliferation, cell migration, mucus secretion, activation of vascular permeability, and regulation of mucosal homeostasis^23–25^. Eosinophils are widely observed in the endometrial stroma, the luminal and glandular epithelium, and the endometrial-myometrial junction of female genital tracts. However, their biological roles are still not well understood. A previous study reported that the presence of eosinophils in endometrial biopsies might indicate chronic endometritis, as well as disordered proliferative endometrium and EPs^26^. Another study suggested that the IL-4 released by eosinophils can promote endometrial stromal cell proliferation and repair genital tissue after infection^27^. Furthermore, elevated eosinophil counts are also frequently observed in patients with nasal polyps, suggesting that eosinophilic inflammation might cause specific mucosal polyps^28–31^. Our study provided some different evidence that higher level of eosinophils had a protective effect on EPs. While up to now, there was no clear evidence regarding the effect of eosinophils on EPs. The results could also vary depending on the source of tissue in the sample being measured. Anyway, our study together with previous studies indicated that eosinophils might be involved in EP pathogenesis.

Our study had several strengths. First, the MR study design not only provided evidences for causal relationships between HBCs and uterine polyps, but also prevented the widespread bias that is common in observational epidemiological studies. Second, the datasets for HBCs and outcomes were all generated from a European population, which avoided the potential bias that might be caused by differences in genetic backgrounds. Third, the large sample size of the GWAS on HBCs guaranteed the strength of the IVs (*F* statistic > 10) used to detect the relationships between HBCs and uterine polyps. All generated IVs were strong instruments for MR analyses.

There were also limitations. First, although MR is a powerful tool for inferring causality, the results should be further verified by experimental studies, and the mechanisms behind the pathogenesis of EPs and CP should be further explored. Second, the study samples of outcomes were limited to females. Gender differences between datasets of exposures and outcomes might introduce bias to the MR estimates. Third, the sample sizes for EPs and CPs were relatively small, more data should be collected to increase the statistical power. Additionally, we did not investigate the associations of the IVs with potential confounders in the two-sample MR estimates.

## Conclusion

The present MR study found that decreased levels of eosinophils were causally associated with a higher risk of EPs. By identifying possible biomarkers for uterine polyps, our study provides novel insight into the pathogenesis of EPs. Our findings may be used to inform clinical diagnostic procedures and future uterine polyp biomarker studies.

## Methods

### Identifying genetic instruments for the 36 HBC traits

The overall flow diagram of this MR study is illustrated in Fig. 1. We used the findings from a large GWAS on 173,480 European-ancestry participants to identify IVs for the 36 HBC traits^32^. The total study samples were composed of three large-scale UK studies, which respectively were 87,265 individuals from the UK Biobank^33^, 45,694 individuals from the UK BiLEVE (a selected subset of the UK Biobank cohort)^34^, and 40,521 individuals form the INTERVAL^35^. HBC traits were measured using clinical hematology analyzers at the centralized processing laboratory of the UK Biocenter (Stockport, UK). Genotyping was performed on the Affymetrix GeneTitan Multi-Channel (MC) Instrument according to the Affymetrix axiom 2.0 assay Automated Workflow. Detailed information for genotype imputation, quality control, and association analysis can be found in a previously published study^32^. Finally, a total of 6736 conditionally independent trait-variant pairs (corresponding to 3755 conditional lead variants) with significance level at *P* < 8.31 × 10^−9^ (a threshold estimated for genome-wide analyses of common, low frequency and rare variants) were identified to compose IVs for the 36 HBC traits. The identified IVs were further mapped to the GWAS datasets of outcomes and SNPs were dropped while not available in datasets of outcomes. The strength of the IVs were evaluated by tow parameters: the proportion of variance explained (R^2^), which was calculated using the formula 2× MAF × (1 − MAF) × (β estimate in SD units)^2^, and the F statistic, which could be calculated from the R^2^ statistic as F = (N – K − 1)/K × R^2^/(1 − R^2^), where N is the sample size and K is the number of SNPs^36^. Typically, a threshold of *F* > 10 is recommended for defining instrument strength in an MR analysis^37^.

### GWAS of EPs and CPs

Genetic associations with EPs and CPs were obtained from the Michigan PheWeb v1.1.17 (http://pheweb.sph.umich.edu/UKBiobank)^38^. The total study sample comprised 7910 EP cases and 3450 CP cases, as well as 396,384 shared heathy controls from the UK Biobank. Cases were diagnosed according to the International Classification of Disease (ICD) codes. Association analyses were performed using SAIGE (https://github.com/weizhouUMICH/SAIGE/), adjusting for genetic relationship, date of birth, as well as the first four principal components. All the data were extracted from the public domain and thus no ethical approval was required for this study.

### Statistical analysis

MR analyses were performed using the random-effect IVW method. Briefly, the IVW approach makes the fundamental assumption that all included genetic variants are valid IVs. This requires each genetic variant to satisfy three conditions: (i) it is strongly associated with the exposure, (ii) it cannot be associated with any confounders, and (iii) it is associated with the outcome exclusively through the exposure^37^. The IVW method is efficient when all variants satisfy the conditions for IV validity. However, bias occurs if horizontal pleiotropy (referring to a situation in which a variant acts on the outcome through other factors besides the exposure) occurs^39^. To control for widespread horizontal pleiotropy in MR analyses, we further performed three additional MR analyses to serve as sensitivity analyses (MR-Egger, weighted median, and MR-PRESSO). MR-Egger provides consistent estimates even with invalid instruments under the Instrument Strength Independent of Direct Effect (InSIDE) assumption^39^. The weighted median introduces a median-based estimator which tolerated up to 50% of the IVs to be invalid, and provides a consistent estimate of causal relationships^40^. MR-PRESSO is a newly developed method that aims to control for horizontal pleiotropy by detecting and correcting for outliers^41^. We also tested for heterogeneity which could indicate pleiotropic instruments effects using the with the *I*^2^ and Cochran Q statistic, and an *I*^2^ > 25% or Cochran Q-derived p < 0.1 was adopted to declare evidence of heterogeneity^42^. Weak or pleiotropic instruments were detected according to the individual components of Q statistic and a corrected model were performed without these outliers^43^. Multivariable MR analyses were performed by using the random-effect IVW method to adjust for the effect of overlapped instruments with other blood traits. Sub-study MR analyses were also performed to avoid potential bias that might be introduced by sample overlapping, using effect size of IVs respectively from UK Biobank, UK BiLEVE and INTERVAL study cohorts^44^.

All MR analyses were carried out using TwoSampleMR and MVMR packages in R (www.cran.r-project.org). A multiple-testing-adjusted threshold of *P* < 6.94 × 10^–4^ (corrected for the total number of comparisons using the Bonferroni method) was defined as the threshold for declaring statistical significance.

### Ethics approval

The GWAS summary statistics for all traits were extracted from the public domain. Therefore, no ethical approval and consent was required for this study.

## Supplementary Information

Supplementary Table 1.

Supplementary Table 2.

## Data Availability

Full summary statistics for the 36 human blood cell traits are publicly available from http://www.bloodcellgenetics.org. GWAS summary statistics for Eps and CPs were downloader from the Michigan PheWeb v1.1.17 (http://pheweb.sph.umich.edu/UKBiobank).

## Abbreviations

Eps: Endometrial polyps
CPs: Cervical polyps
IV: Instrumental variable
MR: Mendelian randomization
GWAS: Genome-wide association study
HBC: Human blood cell
IVW: Inverse-variance weighted
MR-PRESSO: MR-pleiotropy residual sum and outlier
InSIDE: Instrument strength independent of direct effect
SNP: Single nucleotide polymorphism
WBC#: White blood cell count
EO%: Eosinophil percentage of white cells
BASO%: Basophil percentage of white cells
NEUT%: Neutrophil percentage of white cells
MONO%: Monocyte percentage of white cells
LYMPH%: Lymphocyte percentage of white cells
EO#: Eosinophil count
EO%GRAN: Eosinophil percentage of granulocytes
(EO + BASO)#: Sum eosinophil basophil counts
BASO#: Basophil count
BASO%GRAN: Basophil percentage of granulocytes
(BASO + NEUT)#: Sum basophil neutrophil counts
GRAN#: Granulocyte count
GRAN%MYELOID: Granulocyte percentage of myeloid white cells
MYELOID#: Myeloid white cell count
MONO#: Monocyte count
NEUT#: Neutrophil count
NEUT%GRAN: Neutrophil percentage of granulocytes
(NEUT + EO)#: Sum neutrophil eosinophil counts
LYMPH#: Lymphocyte count
RBC#: Red blood cell count
MCV: Mean corpuscular volume
HCT: Hematocrit
MCH: Mean corpuscular hemoglobin
MCHC: Mean corpuscular hemoglobin concentration
HGB: Hemoglobin concentration
RDW: Red cell distribution width
RET#: Reticulocyte count
RET%: Reticulocyte fraction of red cells
IRF: Immature fraction of reticulocytes
HLSR%: High light scatter reticulocyte percentage of red cells
HLSR#: High light scatter reticulocyte count
PCT: Plateletcrit
PDW: Platelet distribution width
PLT#: Platelet count
MPV: Mean platelet volume

## References

[1] Munro MG (2019). Uterine polyps, adenomyosis, leiomyomas, and endometrial receptivity. Fertil. Steril..

[2] Tanos V (2017). The management of polyps in female reproductive organs. Int. J. Surg..

[3] Dreisler E, Stampe Sorensen S, Ibsen PH, Lose G (2009). Prevalence of endometrial polyps and abnormal uterine bleeding in a Danish population aged 20–74 years. Ultrasound Obstet. Gynecol..

[4] Levy RA, Kumarapeli AR, Spencer HJ, Quick CM (2016). Cervical polyps: Is histologic evaluation necessary?. Pathol. Res. Pract..

[5] Nijkang NP, Anderson L, Markham R, Manconi F (2019). Endometrial polyps: Pathogenesis, sequelae and treatment. SAGE Open Med..

[6] DeWaay DJ, Syrop CH, Nygaard IE, Davis WA, Van Voorhis BJ (2002). Natural history of uterine polyps and leiomyomata. Obstet. Gynecol..

[7] Al Chami A, Saridogan E (2017). Endometrial polyps and subfertility. J. Obstet. Gynaecol. India.

[8] de Rijk SR, Steenbergen ME, Nieboer TE, Coppus SF (2016). Atypical endometrial polyps and concurrent endometrial cancer: A systematic review. Obstet. Gynecol..

[9] Berzolla CE (2007). Dysplasia and malignancy in endocervical polyps. J. Womens Health (Larchmt).

[10] Liu Y, Zhang Y, Fu J, Tan W (2012). Inflammation-related gene expression profiles of endocervical polyps. J. Interferon. Cytokine Res..

[11] Troncon JK (2017). Analysis of differential genetic expression in endometrial polyps of postmenopausal women. Climacteric.

[12] Jensen FB (2009). The dual roles of red blood cells in tissue oxygen delivery: Oxygen carriers and regulators of local blood flow. J. Exp. Biol..

[13] van der Meijden PEJ, Heemskerk JWM (2019). Platelet biology and functions: New concepts and clinical perspectives. Nat. Rev. Cardiol..

[14] Buttari B, Profumo E, Rigano R (2015). Crosstalk between red blood cells and the immune system and its impact on atherosclerosis. Biomed. Res. Int..

[15] Jenne CN, Urrutia R, Kubes P (2013). Platelets: Bridging hemostasis, inflammation, and immunity. Int. J. Lab. Hematol..

[16] Friedl P, Weigelin B (2008). Interstitial leukocyte migration and immune function. Nat. Immunol..

[17] Cakmak B (2015). Neutrophil-lymphocyte and platelet-lymphocyte ratios in endometrial hyperplasia. Obstet. Gynecol. Sci..

[18] den Ouden M, Ubachs JM, Stoot JE, van Wersch JW (1997). Whole blood cell counts and leucocyte differentials in patients with benign or malignant ovarian tumours. Eur. J. Obstet. Gynecol. Reprod. Biol..

[19] Davey Smith G, Hemani G (2014). Mendelian randomization: Genetic anchors for causal inference in epidemiological studies. Hum. Mol. Genet..

[20] Lawlor DA, Harbord RM, Sterne JA, Timpson N, Davey Smith G (2008). Mendelian randomization: Using genes as instruments for making causal inferences in epidemiology. Stat. Med..

[21] Zheng J (2017). Recent developments in mendelian randomization studies. Curr. Epidemiol. Rep..

[22] Rothenberg ME, Hogan SP (2006). The eosinophil. Annu. Rev. Immunol..

[23] Schmid-Grendelmeier P (2002). Eosinophils express functional IL-13 in eosinophilic inflammatory diseases. J. Immunol..

[24] Heredia JE (2013). Type 2 innate signals stimulate fibro/adipogenic progenitors to facilitate muscle regeneration. Cell.

[25] Shah K, Ignacio A, McCoy KD, Harris NL (2020). The emerging roles of eosinophils in mucosal homeostasis. Mucosal Immunol..

[26] Adegboyega PA, Pei Y, McLarty J (2010). Relationship between eosinophils and chronic endometritis. Hum. Pathol..

[27] Vicetti Miguel RD (2017). IL-4-secreting eosinophils promote endometrial stromal cell proliferation and prevent Chlamydia-induced upper genital tract damage. Proc. Natl. Acad. Sci. U. S. A..

[28] Shah SA, Ishinaga H, Takeuchi K (2016). Pathogenesis of eosinophilic chronic rhinosinusitis. J. Inflamm. (Lond.).

[29] Khalmuratova R (2018). Wogonin attenuates nasal polyp formation by inducing eosinophil apoptosis through HIF-1alpha and survivin suppression. Sci. Rep..

[30] Tecimer SH (2015). Correlation between clinical findings and eosinophil/neutrophil ratio in patients with nasal polyps. Eur. Arch. Otorhinolaryngol..

[31] Brescia G (2020). Prognostic role of blood eosinophil and basophil levels in allergic fungal rhinosinusitis (AFRS). Am. J. Otolaryngol..

[32] Astle WJ (2016). The allelic landscape of human blood cell trait variation and links to common complex disease. Cell.

[33] Sudlow C (2015). UK biobank: An open access resource for identifying the causes of a wide range of complex diseases of middle and old age. PLoS Med..

[34] Wain LV (2015). Novel insights into the genetics of smoking behaviour, lung function, and chronic obstructive pulmonary disease (UK BiLEVE): A genetic association study in UK Biobank. Lancet Respir. Med..

[35] Moore C (2014). The INTERVAL trial to determine whether intervals between blood donations can be safely and acceptably decreased to optimise blood supply: Study protocol for a randomised controlled trial. Trials.

[36] Burgess S, Dudbridge F, Thompson SG (2016). Combining information on multiple instrumental variables in Mendelian randomization: Comparison of allele score and summarized data methods. Stat. Med..

[37] Burgess S, Butterworth A, Thompson SG (2013). Mendelian randomization analysis with multiple genetic variants using summarized data. Genet. Epidemiol..

[38] Zhou W (2018). Efficiently controlling for case-control imbalance and sample relatedness in large-scale genetic association studies. Nat. Genet..

[39] Bowden J, Davey Smith G, Burgess S (2015). Mendelian randomization with invalid instruments: Effect estimation and bias detection through Egger regression. Int. J. Epidemiol..

[40] Bowden J, Davey Smith G, Haycock PC, Burgess S (2016). Consistent estimation in mendelian randomization with some invalid instruments using a weighted median estimator. Genet. Epidemiol..

[41] Verbanck M, Chen CY, Neale B, Do R (2018). Detection of widespread horizontal pleiotropy in causal relationships inferred from Mendelian randomization between complex traits and diseases. Nat. Genet..

[42] Greco MF, Minelli C, Sheehan NA, Thompson JR (2015). Detecting pleiotropy in Mendelian randomisation studies with summary data and a continuous outcome. Stat. Med..

[43] Havdahl A, Mitchell R, Paternoster L, Davey Smith G (2019). Investigating causality in the association between vitamin D status and self-reported tiredness. Sci. Rep..

[44] Burgess S, Davies NM, Thompson SG (2016). Bias due to participant overlap in two-sample Mendelian randomization. Genet. Epidemiol..

