# Supplementary material for: Mendelian randomization analysis of the association between human blood cell traits and uterine polyps
Source: Sci Rep. 2021 Mar 4;11:5234. doi: 10.1038/s41598-021-84851-0 (PMC7933156; doi:10.1038/s41598-021-84851-0)
Supplement: Supplementary file 1 — Supplementary Table 1. [file 41598_2021_84851_MOESM1_ESM.docx]

**Mendelian randomization analysis of the association between human blood cell traits** **and** **uterine** **polyps**

Shuliu Sun^1#^, Yan Liu^1#^, Lanlan Li^1^, Minjie Jiao^1^, Yufen Jiang^1^, Beilei Li^1^, Wenrong Gao^1^, Xiaojuan Li^1^

^1^ Department of Obstetrics and Gynecology, Northwest Women's and Children's Hospital, Xi'an, China

^#^ These authors contributed equally to this work.

**Correspondence to:** Xiaojuan Li, MD

Department of Obstetrics and Gynecology, Northwest Women's and Children's Hospital, Xi'an, Shaanxi 710061, China; Phone: +86 89622139.

Email: [lxjuann@outlook.com](mailto:lxjuann@outlook.com)

**Supplementary Table 1. Strength of the instrumental variables used as genetic predictors for human blood cell traits**

| **Cell Type** | **HBC Traits** | **Full Name** | **#SNPs** | **Explained R^2^** | **F statistic** |
| --- | --- | --- | --- | --- | --- |
| Compound white cell | WBC# | White blood cell count | 135 | 7.7% | 106.4 |
|  | EO% | Eosinophil percentage of white cells | 156 | 8.8% | 107.0 |
|  | BASO% | Basophil percentage of white cells | 52 | 2.8% | 95.3 |
|  | NEUT% | Neutrophil percentage of white cells | 102 | 4.4% | 78.4 |
|  | MONO% | Monocyte percentage of white cells | 187 | 16.6% | 183.9 |
|  | LYMPH% | Lymphocyte percentage of white cells | 104 | 4.4% | 75.9 |
| Myeloid white cell | EO# | Eosinophil count | 167 | 9.7% | 111.7 |
|  | EO%GRAN | Eosinophil percentage of granulocytes | 155 | 8.5% | 104.5 |
|  | (EO+BASO)# | Sum eosinophil basophil counts | 164 | 9.2% | 107.1 |
|  | BASO# | Basophil count | 68 | 3.1% | 82.4 |
|  | BASO%GRAN | Basophil percentage of granulocytes | 46 | 2.8% | 107.0 |
|  | (BASO+NEUT)# | Sum basophil neutrophil counts | 113 | 6.3% | 103.1 |
|  | GRAN# | Granulocyte count | 116 | 6.6% | 105.2 |
|  | GRAN%MYELOID | Granulocyte percentage of myeloid white cells | 173 | 13.7% | 158.9 |
|  | MYELOID# | Myeloid white cell count | 122 | 7.6% | 116.7 |
|  | MONO# | Monocyte count | 191 | 16.4% | 178.0 |
|  | NEUT# | Neutrophil count | 114 | 6.3% | 102.0 |
|  | NEUT%GRAN | Neutrophil percentage of granulocytes | 139 | 7.6% | 102.9 |
|  | (NEUT+EO)# | Sum neutrophil eosinophil counts | 116 | 6.6% | 105.0 |
| Lymphoid white cell | LYMPH# | Lymphocyte count | 156 | 9.1% | 110.7 |
| Mature red cell | RBC# | Red blood cell count | 164 | 11.1% | 131.7 |
|  | MCV | Mean corpuscular volume | 246 | 24.3% | 225.7 |
|  | HCT | Hematocrit | 106 | 5.8% | 100.4 |
|  | MCH | Mean corpuscular hemoglobin | 215 | 24.5% | 261.8 |
|  | MCHC | Mean corpuscular hemoglobin concentration | 69 | 5.7% | 152.2 |
|  | HGB | Hemoglobin concentration | 118 | 6.5% | 101.7 |
|  | RDW | Red cell distribution width | 168 | 12.6% | 149.2 |
| Immature red cell | RET# | Reticulocyte count | 165 | 14.0% | 170.6 |
|  | RET% | Reticulocyte fraction of red cells | 174 | 14.8% | 172.5 |
|  | IRF | Immature fraction of reticulocytes | 117 | 9.9% | 162.3 |
|  | HLSR% | High light scatter reticulocyte percentage of red cells | 154 | 12.8% | 165.8 |
|  | HLSR# | High light scatter reticulocyte count | 170 | 13.4% | 157.5 |
| Platelet | PCT | Plateletcrit | 209 | 15.9% | 156.5 |
|  | PDW | Platelet distribution width | 169 | 15.2% | 183.7 |
|  | PLT# | Platelet count | 224 | 17.8% | 167.7 |
|  | MPV | Mean platelet volume | 242 | 28.3% | 281.9 |
